# Supplementary material for: Saffold virus exploits integrin αvβ8 and sulfated glycosaminoglycans as cooperative attachment receptors for infection
Source: Nat Commun. 2025 Dec 15;17:534. doi: 10.1038/s41467-025-67236-z (PMC12804938; doi:10.1038/s41467-025-67236-z)
Supplement: Supplementary file 1 — Supplementary Information [file 41467_2025_67236_MOESM1_ESM.pdf]

Supplementary Information for

Saffold Virus Exploits Integrin $\alpha V\beta 8$ and Sulfated Glycosaminoglycans as Cooperative Attachment Receptors for Infection

Takako Okuwa [1,*], Toshiki Himeda [1,*,**], Kyousuke Kobayashi [2,*], Namiko Nomura [2], Kouichi Utani [1], Satoshi Koike [2], Akira Nakamura [3], Masaya Higuchi [1,**]

[1] Department of Microbiology, Kanazawa Medical University School of Medicine, Ishikawa, Japan
[2] Neurovirology Project, Department of Genome Medicine, Tokyo Metropolitan Institute of Medical Science, Tokyo, Japan
[3] Division of Immunology, Faculty of Medicine, Tohoku Medical and Pharmaceutical University, Miyagi, Japan

*TO, TH and KK contributed equally to this work.

**Co-corresponding author:
Toshiki HIMEDA, PhD
E-mail: himeda@kanazawa-med.ac.jp
Masaya HIGUCHI, M.D., Ph.D., Professor and Chairman
E-mail: masahigu@kanazawa-med.ac.jp

Supplementary Figures 1-9
Supplementary Table 1

a

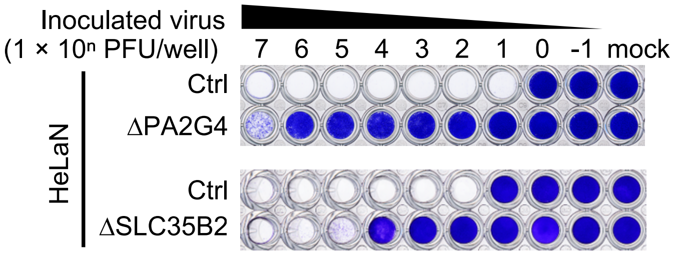

Gene	sgRNA sequence
PA2G4	CAGTGACCTTGGGGTCCATG
SLC35B2	AGAGTGATGACCCGCAGCTA

b

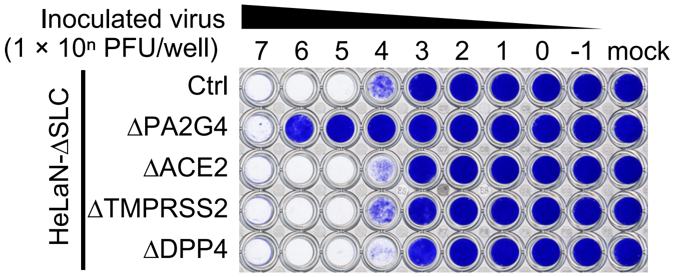

Gene	sgRNA sequence
ACE2	ATGAGCACCATCTACAGTAC
TMPRSS2	CGGATGCACCTCGTAGACAG
DPP4	ACAACACACAGTGGGTCACA

Supplementary Fig. 1. Validation of hit genes.

The validation experiments were conducted on hit genes identified in the primary screening (**a**) and in the secondary screening except for *ITGAV* and *ITGB8* (**b**), using bulk KO cell populations. Control HeLa-N cells and the indicated KO cell populations were infected with 10-fold serial dilutions of SAFV-3, and viable cells were stained with crystal violet. sgRNA sequences used for gene KO are listed in the right panels. Images are representative of two independent experiments. PA2G4: proliferation-associated 2G4, ACE2: angiotensin converting enzyme 2: dipeptidyl peptidase 4, TMPRSS2: transmembrane serine protease 2, DPP4: dipeptidyl peptidase 4

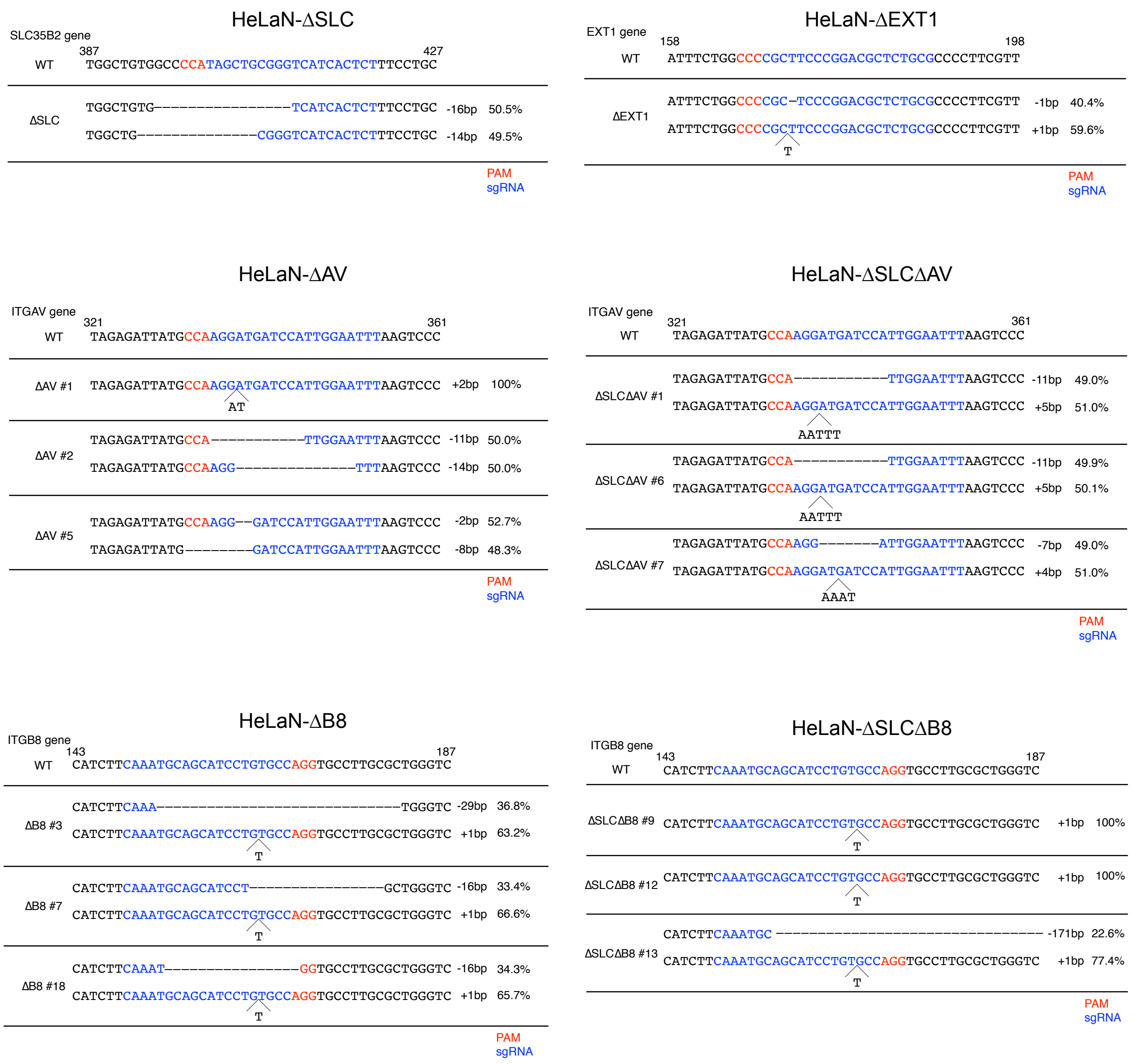

Supplementary Fig. 2. Alignment of nucleic acid sequences at sgRNA target sites in clonal KO cells.

Nucleotide sequence from HeLaN-ΔSLC, HeLaN-ΔEXT1, HeLaN-ΔAV, HeLaN-ΔSLCΔAV, HeLaN-ΔB8, and HeLaN-ΔSLCΔB8 cells were aligned against those from HeLaN-WT cells. sgRNA target sites are highlighted in blue. Dashed lines indicate deleted bases in KO cells, and black "^" symbols represent inserted bases. Protospacer adjacent motif (PAM) sites are shown in red.

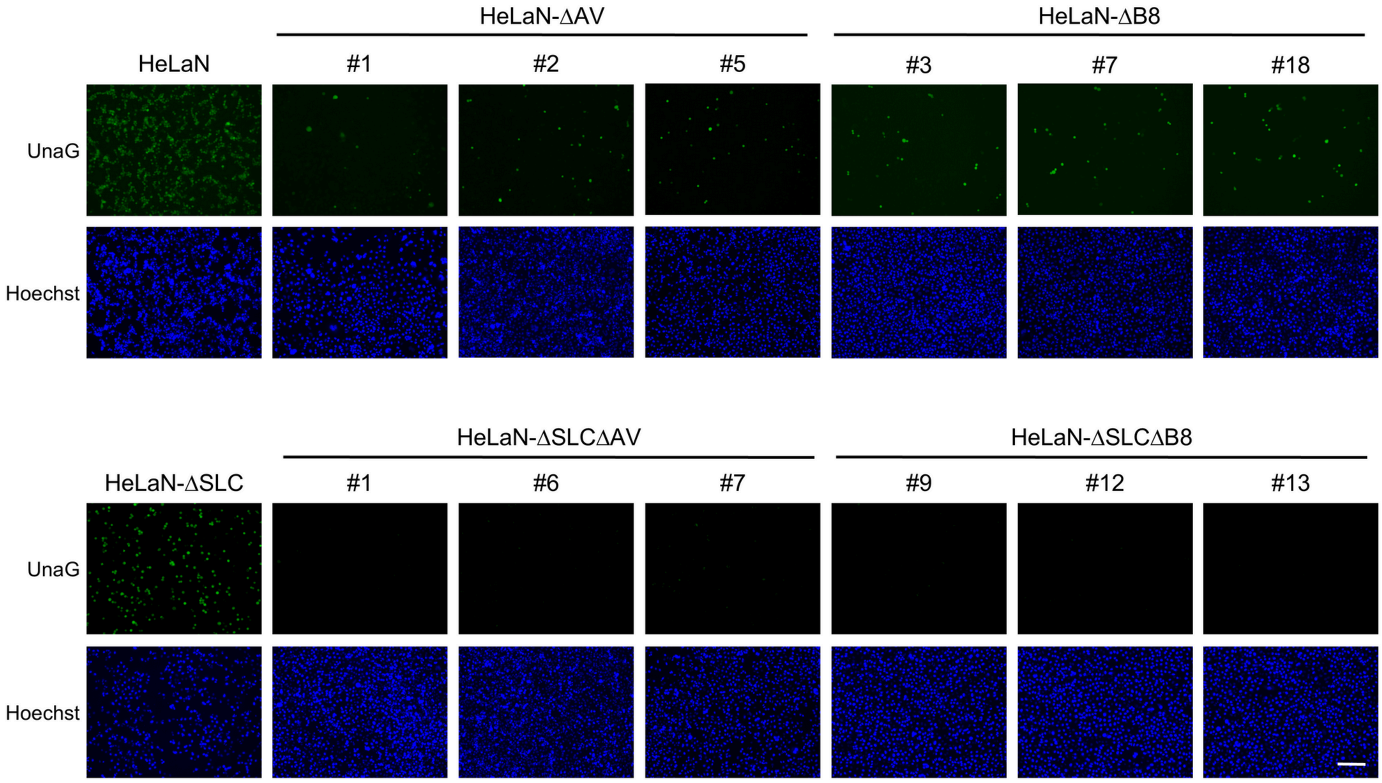

Supplementary Fig. 3. Susceptibility analysis using SAF/UnaG in HeLaN-ΔAV, HeLaN-ΔSLCΔAV, HeLaN-ΔB8, and HeLaN-ΔSLCΔB8 clonal cells.

UnaG-positive cells (green, upper panel) and nuclei stained with Hoechst (blue, lower panel) were imaged at 16 hours post-infection. Scale bar, 200 μm. Images are representative of two independent experiments.

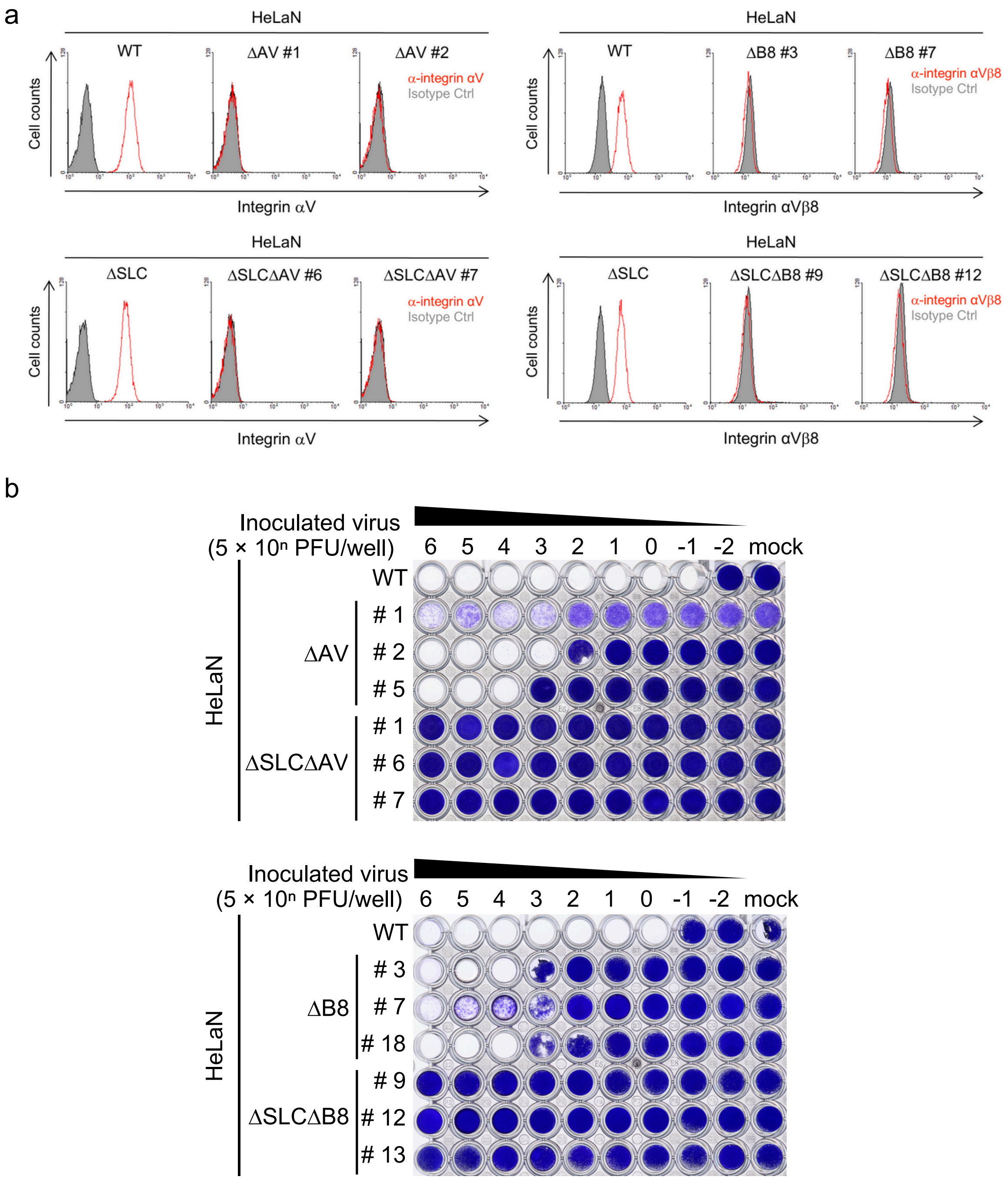

Supplementary Fig. 4. Characterization of *ITGAV* and *ITGB8* clonal KO cells.

a, Expression of human integrin αV and integrin β8 in HeLaN-ΔAV, HeLaN-ΔSLCΔAV, HeLaN-ΔB8, and HeLaN-ΔSLCΔB8 clonal cells. Cells were stained with anti-integrin αV or anti-integrin αVβ8 antibodies and analyzed by flow cytometry. **b**, The same clonal cell lines were infected with 10-fold serial dilutions of SAFV-3 and incubated for 5 days. Viable cells were then stained with crystal violet to assess infection levels. Images are representative of two independent experiments.

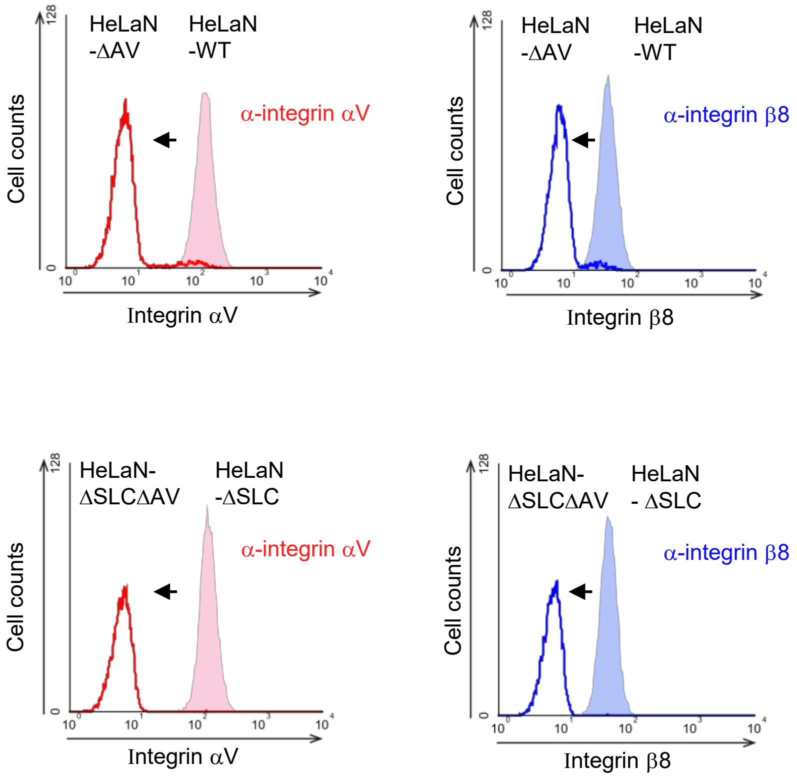

Supplementary Fig. 5. Cell surface expression of integrin β8 in *ITGAV* KO cells.

Overlay histograms showing the expression of integrin αV and β8 in HeLaN-ΔAV compared to HeLaN-WT (upper panels), and in HeLaN-ΔSLCΔAV compared to HeLaN-ΔSLC (lower panels). Cells were stained with the indicated antibodies and analyzed by flow cytometry. For the detection of cell surface integrin β8, rabbit anti-human integrin β8 antibody Clone # 2723C (MAB47752, R&D systems) was used.

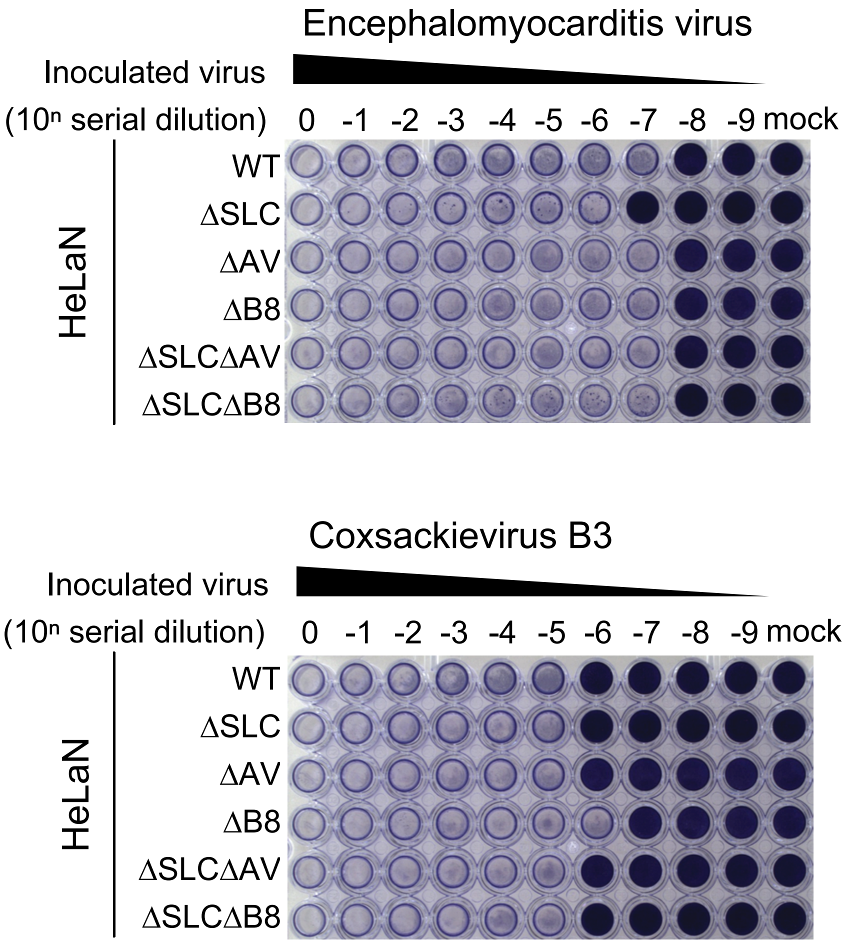

Supplementary Fig. 6. Validation of SAFV-specific resistance in KO cell lines used in this study.

Clonal KO cell lines for *ITGAV*, *ITGB8*, and/or *SLC35B2* were infected with 10-fold serial dilutions of encephalomyocarditis virus (upper panel) and Coxsackievirus B3 (lower panel). Viable cells were stained with crystal violet to assess infection susceptibility, confirming that resistance was specific to SAFV.

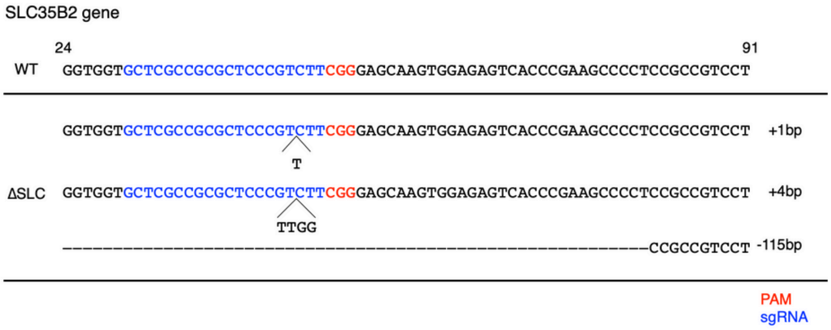

Supplementary Fig. 7. Alignment of nucleic acid sequences at sgRNA target sites in clonal BHK-ΔSLC cells compared to WT BHK-21 cells.

sgRNA target sites are highlighted in blue. Dashed lines indicate deleted bases in KO cells, and black “^” symbols represent inserted bases. PAM sites are shown in red.

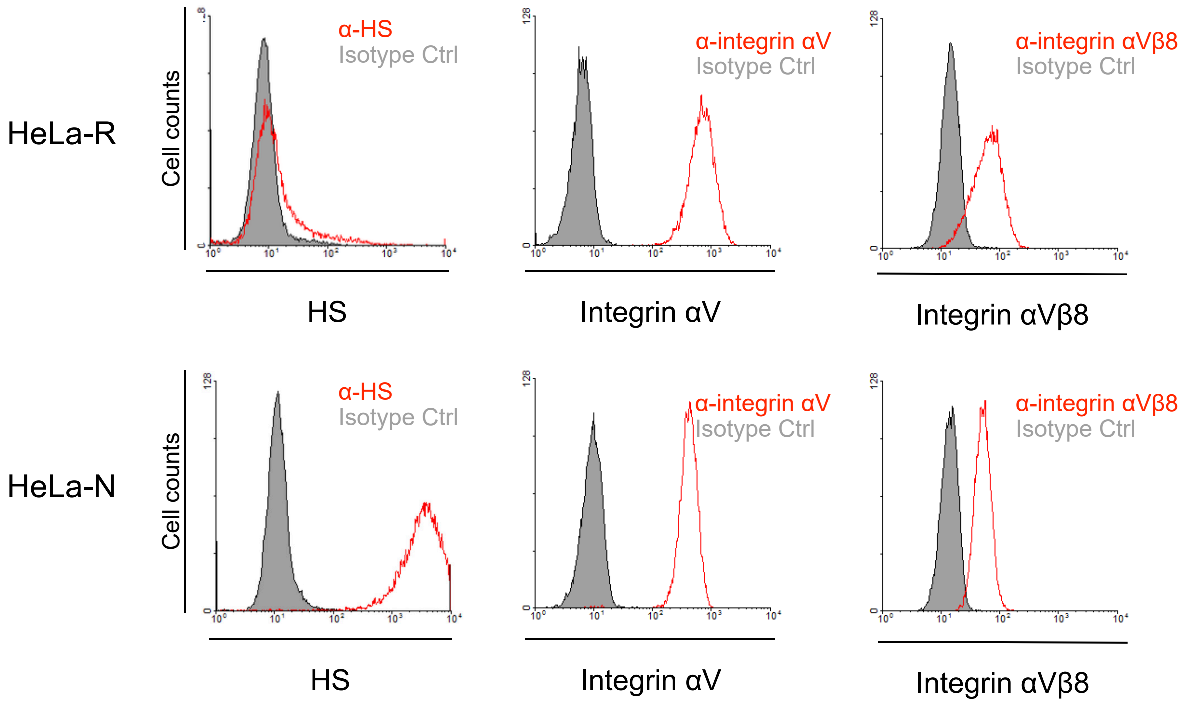

Supplementary Fig. 8. Expression of HS and human integrin αVβ8 in HeLa-N and HeLa-R cells.

HeLa-R (upper panel) and HeLa-N (lower panel) cells were stained with the indicated antibodies and analyzed by flow cytometry.

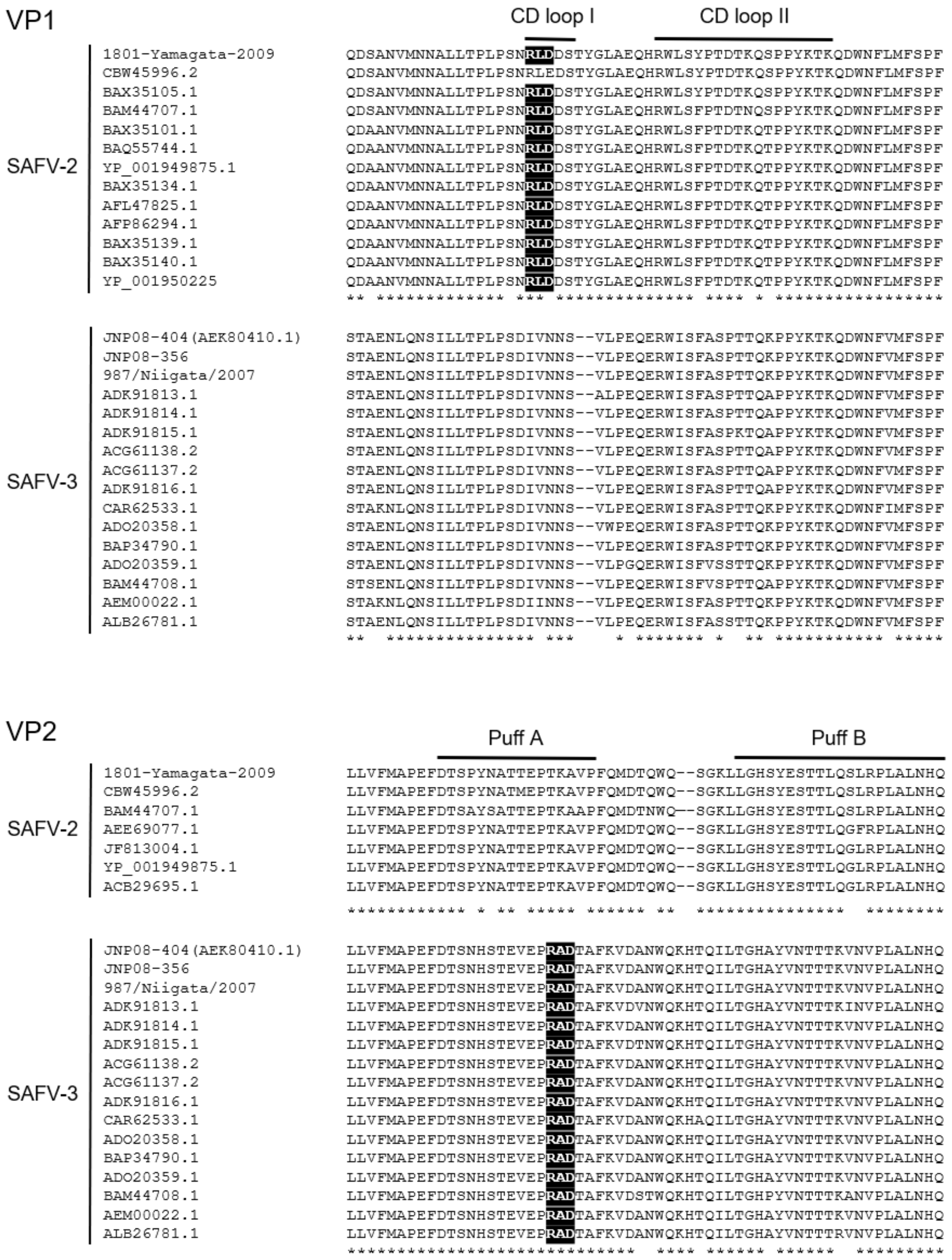

Supplementary Fig. 9. RGD-like sequences on VP1 of SAFV-2 and VP2 of SAFV-3.

Amino acid sequence alignment of CD loops I and II on VP1 (upper panel) and puffs A and B on VP2 (lower panel) of SAFV-2 and SAFV-3. RGD-like sequences are highlighted in black.

Supplementary Table 1. Summary of passage numbers and cell lines used for isolation of clinical strains.

			Cells for isolation		Propagation for this study	
Strain	Genotype	Accession No.	Cell line	Passage number	Cell line	Passage number
JPN08-356	3	LC865996	LLC-MK2	not documented	HeLa-N	2
987/Niigata/2007	3	LC460463	1. LLC-MK2<br>2. RD-18S-N	2<br>2	HeLa-N	2
1801-Yamagata-2009	2	LC865997	RD-18S-N	4	RD-18S-N	2

The passage number of each clinical strain along with the corresponding cell lines used for their isolation are shown. The characteristics of HeLa-N and RD-18S-N cell lines are described in References 19 and 56, respectively.
