# Supplementary material for: Saffold virus exploits integrin αvβ8 and sulfated glycosaminoglycans as cooperative attachment receptors for infection
Source: Nat Commun. 2025 Dec 15;17:534. doi: 10.1038/s41467-025-67236-z (PMC12804938; doi:10.1038/s41467-025-67236-z)
Supplement: Supplementary file 2 — Reporting summary [file 41467_2025_67236_MOESM2_ESM.pdf]

nature portfolio

Corresponding author(s): Toshiki HIMEDA
Masaya HIGUCHI

Last updated by author(s): Nov 21, 2025

Reporting Summary

Nature Portfolio wishes to improve the reproducibility of the work that we publish. This form provides structure for consistency and transparency in reporting. For further information on Nature Portfolio policies, see our Editorial Policies and the Editorial Policy Checklist.

Statistics

For all statistical analyses, confirm that the following items are present in the figure legend, table legend, main text, or Methods section.

n/a	Confirmed	
☐	☒	The exact sample size (*n*) for each experimental group/condition, given as a discrete number and unit of measurement
☐	☒	A statement on whether measurements were taken from distinct samples or whether the same sample was measured repeatedly
☐	☒	The statistical test(s) used AND whether they are one- or two-sided<br>*Only common tests should be described solely by name; describe more complex techniques in the Methods section.*
☒	☐	A description of all covariates tested
☒	☐	A description of any assumptions or corrections, such as tests of normality and adjustment for multiple comparisons
☐	☒	A full description of the statistical parameters including central tendency (e.g. means) or other basic estimates (e.g. regression coefficient) AND variation (e.g. standard deviation) or associated estimates of uncertainty (e.g. confidence intervals)
☐	☒	For null hypothesis testing, the test statistic (e.g. *F*, *t*, *r*) with confidence intervals, effect sizes, degrees of freedom and *P* value noted<br>*Give P values as exact values whenever suitable.*
☒	☐	For Bayesian analysis, information on the choice of priors and Markov chain Monte Carlo settings
☒	☐	For hierarchical and complex designs, identification of the appropriate level for tests and full reporting of outcomes
☒	☐	Estimates of effect sizes (e.g. Cohen's *d*, Pearson's *r*), indicating how they were calculated

Our web collection on statistics for biologists contains articles on many of the points above.

Software and code

Policy information about availability of computer code

Data collection	No software was used.
Data analysis	GraphPad Prism software program version 7,<br>ImageJ software program version 1.48,<br>WinMDI software program version 2.9,<br>Kaluza software program version 2.3.1,<br>Prinseq software program, version 0.20.4,<br>TIDE version 3.3.0,<br>MAGeCK version 0.5.9.5

For manuscripts utilizing custom algorithms or software that are central to the research but not yet described in published literature, software must be made available to editors and reviewers. We strongly encourage code deposition in a community repository (e.g. GitHub). See the Nature Portfolio guidelines for submitting code & software for further information.

Data

Policy information about availability of data

All manuscripts must include a data availability statement. This statement should provide the following information, where applicable:

- Accession codes, unique identifiers, or web links for publicly available datasets
- A description of any restrictions on data availability
- For clinical datasets or third party data, please ensure that the statement adheres to our policy

Genome sequences of JPN08-404 strain (GenBank accession no. HQ902242), JPN08-356 strain (DDBJ accession no. LC865996), 987/Niigata/2007 (DDBJ accession no. LC460463), and 1801-Yamagata-2009 (DDBJ accession no. LC865997) can be accessed through the NCBI Nucleotide database.
The raw sequencing data from the CRISPR screens (accession number PRJDB20430) can be accessed through the DDBJ BioProject.

GenBank accession numbers are as follows:
Human SLC35B2, NM_178148
Hamster SLC35B2, XP_005072381.1
Human EXT1, NM_000127.3
Human ITGAV, NP_002201.2
Human ITGB8, NM_002214.3
Mouse ITGB8, NM_177290.4
Hamster ITGB8, XM_005084467
Human ITGB1, NM_002211.4
Human ITGB3, NM_000212.3
Human ITGB5, NM_002213.5
Human ITGB6, NM_000888.5

Research involving human participants, their data, or biological material

Policy information about studies with human participants or human data. See also policy information about sex, gender (identity/presentation), and sexual orientation and race, ethnicity and racism.

Reporting on sex and gender	Not applicable
Reporting on race, ethnicity, or other socially relevant groupings	Not applicable
Population characteristics	Not applicable
Recruitment	Not applicable
Ethics oversight	Not applicable

Note that full information on the approval of the study protocol must also be provided in the manuscript.

Field-specific reporting

Please select the one below that is the best fit for your research. If you are not sure, read the appropriate sections before making your selection.

☒ Life sciences ☐ Behavioural & social sciences ☐ Ecological, evolutionary & environmental sciences

For a reference copy of the document with all sections, see nature.com/documents/nr-reporting-summary-flat.pdf

Life sciences study design

All studies must disclose on these points even when the disclosure is negative.

Sample size	Sample sizes were estimated on the basis of previous studies using similar methods, see Yamayoshi et al. (DOI: 10.1038/nm.1992); Watanabe et al. (DOI: 10.1038/s41467-023-37399-8.)
Data exclusions	No data were excluded from the analyses.
Replication	All experiments were repeated at least twice and yielded similar results.
Randomization	Not relevant to this study, since samples were not allocated into experimental groups.
Blinding	No blinding was performed in this study, because there is no clinical data or field sample collection.

April 2023

Reporting for specific materials, systems and methods

We require information from authors about some types of materials, experimental systems and methods used in many studies. Here, indicate whether each material, system or method listed is relevant to your study. If you are not sure if a list item applies to your research, read the appropriate section before selecting a response.

Materials & experimental systems

n/a	Involved in the study
☐	☒ Antibodies
☐	☒ Eukaryotic cell lines
☒	☐ Palaeontology and archaeology
☒	☐ Animals and other organisms
☒	☐ Clinical data
☒	☐ Dual use research of concern
☒	☐ Plants

Methods

n/a	Involved in the study
☒	☐ ChIP-seq
☐	☒ Flow cytometry
☒	☐ MRI-based neuroimaging

Antibodies

Antibodies used

Biotinylated mouse anti-heparan sulfate antibody (10E4 epitope) (370255-B, amsbio)
Biotinylated mouse IgMκ Isotype control (401621, BioLegend)
PE-streptavidin (405203, BioLegend)
PE-conjugated antibodies against CD51 (integrin αV) (327910, BioLegend)
CD29 (integrin β1) (303003, BioLegend)
CD61 (integrin β3) (336405, BioLegend)
Integrin β5 (345203, BioLegend)
APC-conjugated antibody against integrin β6 (FAB4155A, R&D Systems)
PE-conjugated mouse IgG1κ (981804, BioLegend) isotype
PE-conjugated IgG2aκ (400213, BioLegend) isotype
Rabbit anti-integrin αVβ8 (clone EM13309) (ZRB1192, Sigma Aldrich)
PE-conjugated donkey anti-rabbit IgG secondary antibody (406421, BioLegend)
Rabbit Polyclonal Isotype antibody (910801, BioLegend)
Rabbit anti-Integrin αV polyclonal antibody (27096-1-AP, Proteintech)
Rabbit anti-integrin β8 (D1V7M) monoclonal antibody (88300, Cell Signaling Technology)
Rabbit-anti-integrin αVβ3 monoclonal antibody (clone EM22703), (ZRB1190, Sigma-Aldrich)
mouse anti-actin (AC-40) monoclonal antibody (A3853, Sigma Aldrich)
horseradish peroxidase-conjugated anti-mouse IgG (170-6516, Bio-Rad Laboratories)
horseradish peroxidase-conjugated anti-rabbit IgG (170-6515, Bio-Rad Laboratories)
rabbit anti-human integrin β8 antibody Clone # 2723C (MAB47752, R&D systems)
rabbit anti-SAFV-3 antiserum

Validation

Commercial antibodies were validated by the suppliers, we refer to the information on the supplier's websites.
Rabbit anti-SAFV-3 antiserum was used in our previous study (Himeda et al. doi:10.1371/ journal.pone.0053194).

Eukaryotic cell lines

Policy information about cell lines and Sex and Gender in Research

Cell line source(s)

HeLa-R RIKEN BRC (RCB0007) (DOI: 10.1371/journal.pone.0053194)
HeLa-N (DOI: 10.1371/journal.pone.0053194)
293T (DOI: 10.1128/JVI.00532-07)
BHK-21 (DOI: 10.1128/JVI.02385-08)
BHK-21 (C-13) JCRB Cell Bank (JCRB9020)
Caco-2 (DOI: 10.3201/eid1306.060896)
RD-18S-Niigata (DOI: 10.1002/jmv.24928)

Authentication

HeLa-N and HeLa-R cell lines were authenticated by STR analysis (DOI: 10.1371/journal.pone.0053194).
Other cell lines were not authenticated.

Mycoplasma contamination

All cell lines were not tested for mycoplasma contamination.

Commonly misidentified lines
(See ICLAC register)

No commonly misidentified cell lines were used.

Plants

Seed stocks	*Report on the source of all seed stocks or other plant material used. If applicable, state the seed stock centre and catalogue number. If plant specimens were collected from the field, describe the collection location, date and sampling procedures.*
Novel plant genotypes	*Describe the methods by which all novel plant genotypes were produced. This includes those generated by transgenic approaches, gene editing, chemical/radiation-based mutagenesis and hybridization. For transgenic lines, describe the transformation method, the number of independent lines analyzed and the generation upon which experiments were performed. For gene-edited lines, describe the editor used, the endogenous sequence targeted for editing, the targeting guide RNA sequence (if applicable) and how the editor was applied.*
Authentication	*Describe any authentication procedures for each seed stock used or novel genotype generated. Describe any experiments used to assess the effect of a mutation and, where applicable, how potential secondary effects (e.g. second site T-DNA insertions, mosiacism, off-target gene editing) were examined.*

Flow Cytometry

Plots

Confirm that:

☐ The axis labels state the marker and fluorochrome used (e.g. CD4-FITC).

☐ The axis scales are clearly visible. Include numbers along axes only for bottom left plot of group (a 'group' is an analysis of identical markers).

☐ All plots are contour plots with outliers or pseudocolor plots.

☐ A numerical value for number of cells or percentage (with statistics) is provided.

Methodology

Sample preparation	~500,000 cells were harvested using Accutase (Nacalai Tesque) and washed in FACS buffer (PBS + 3% FCS). The cells were incubated with primary antibodies for 30 minutes on ice. Cells were washed in FACS buffer and subsequently incubated in secondary antibodies for 30 minutes on ice, if required. Cells were washed and resuspend in FACS buffer. Samples were analyzed using a FACS Canto II (BD Biosciences) and the WinMDI software.
Instrument	FACS Canto II (BD Biosciences)<br>DxFLEX (Beckman Coulter)
Software	WinMDI software version 2.9<br>Kaluza software version 2.3.1
Cell population abundance	N/A
Gating strategy	Living cells were gated based on SSC-A vs. FSC-A plot. Negative controls were prepared using isotype controls or without antibody.

☐ Tick this box to confirm that a figure exemplifying the gating strategy is provided in the Supplementary Information.
